# Effects of Antiepileptic Drugs on Spontaneous Recurrent Seizures in a Novel Model of Extended Hippocampal Kindling in Mice

**DOI:** 10.3389/fphar.2018.00451

**Published:** 2018-05-18

**Authors:** Hongmei Song, Uilki Tufa, Jonathan Chow, Nila Sivanenthiran, Chloe Cheng, Stellar Lim, Chiping Wu, Jiachun Feng, James H. Eubanks, Liang Zhang

**Affiliations:** ^1^Krembil Research Institute, University Health Network, Toronto, ON, Canada; ^2^Department of Neurosurgery, The First Hospital of Jilin University, Changchun, China; ^3^Department of Neurology, The First Hospital of Jilin University, Changchun, China; ^4^Department of Surgery, University of Toronto, Toronto, ON, Canada; ^5^Epilepsy Program, University of Toronto, Toronto, ON, Canada; ^6^Department of Medicine, University of Toronto, Toronto, ON, Canada

**Keywords:** antiepileptic drugs, convulsion, electroencephalograph (EEG), epilepsy, hippocampus, kindling, mice, spontaneous seizures

## Abstract

Epilepsy is a common neurological disorder characterized by naturally-occurring spontaneous recurrent seizures and comorbidities. Kindling has long been used to model epileptogenic mechanisms and to assess antiepileptic drugs. In particular, extended kindling can induce spontaneous recurrent seizures without gross brain lesions, as seen clinically. To date, the development of spontaneous recurrent seizures following extended kindling, and the effect of the antiepileptic drugs on these seizures are not well understood. In the present study we aim to develop a mouse model of extended hippocampal kindling for the first time. Once established, we plan to evaluate the effect of three different antiepileptic drugs on the development of the extended-hippocampal-kindled-induced spontaneous recurrent seizures. Male C57 black mice were used for chronic hippocampal stimulations or handling manipulations (twice daily for up to 70 days). Subsequently, animals underwent continuous video/EEG monitoring for seizure detection. Spontaneous recurrent seizures were consistently observed in extended kindled mice but no seizures were detected in the control animals. The aforementioned seizures were generalized events characterized by hippocampal ictal discharges and concurrent motor seizures. Incidence and severity of the seizures was relatively stable while monitored over a few months after termination of the hippocampal stimulation. Three antiepileptic drugs with distinct action mechanisms were tested: phenytoin, lorazepam and levetiracetam. They were applied via intra-peritoneal injections at anticonvulsive doses and their effects on the spontaneous recurrent seizures were analyzed 10–12 h post-injection. Phenytoin (25 mg/kg) and levetiracetam (400 mg/kg) abolished the spontaneous recurrent seizures. Lorazepam (1.5 mg/kg) decreased motor seizure severity but did not reduce the incidence and duration of corresponding hippocampal discharges, implicating its inhibitory effects on seizure spread. No gross brain lesions were observed in a set of extended hippocampal kindled mice submitted to histological evaluation. All these data suggests that our model could be considered as a novel mouse model of extended hippocampal kindling. Some limitations remain to be considered.

## Introduction

Epilepsy is a common neurological disorder characterized by naturally-occurring spontaneous recurrent seizures (SRS) and comorbidities. Temporal lobe epilepsy (TLE) is the most common type of drug-resistant epilepsy in the adult/aging population (Engel, 1996). Kindling via chronic electrical stimulation of limbic structures has long been used to model TLE, to assess the antiepileptic drugs (AEDs) efficacy and to explore drug-resistant epilepsy (Gorter et al., 2016; Löscher, 2017; Sutula and Kotloski, 2017). While classic kindling over a few weeks does not generally induce spontaneous seizures, extended kindling is able to induce SRS in several animal species. Specifically, extended kindling of the amygdala, hippocampus, perforant path or olfactory bulb induces SRS in monkeys (Wada and Osawa, 1976), dogs (Wauquier et al., 1979), cats (Wada et al., 1974; Gotman, 1984; Hiyoshi et al., 1993) and rats (Pinel and Rovner, 1978; Milgram et al., 1995; Michalakis et al., 1998; Sayin et al., 2003; Brandt et al., 2004). SRS development following extended amygdala kindling is generally associated with a loss of subgroups of hippocampal GABAergic interneurons in dentate gyrus-hilar areas (Sayin et al., 2003; Brandt et al., 2004) rather than gross brain lesions as seen in different status epilepticus models (Dudek and Staley, 2017; Gorter and van Vliet, 2017; Henshall, 2017; Kelly and Coulter, 2017). This way, extended kindling may help model the genesis of SRS without a major brain pathology as usually seen in TLE patients (Ferlazzo et al., 2016). To date, SRS following extended kindling and the effect of different AEDs on such seizures are still not well understood.

Mouse models have been largely used in epilepsy research because the genetic/molecular manipulations done in these animals offer great advantages in mechanistic investigation. However, although several studies have examined classic kindling seizures in mice, no extended kindling mouse model has been established yet. In this sense, we aim to develop such a model to facilitate future ictogenesis research and the possible effects that AEDs might have on SRS. To achieve success, we took into consideration several factors such as the strain-dependent susceptibility to have seizures/epilepsy (Löscher et al., 2017), the reliability of chronic electroencephalography (EEG) in mouse models (Bertram, 2017), and the pharmacokinetics of the AEDs in rodents (faster elimination than in humans) (Löscher, 2007; Markowitz et al., 2010). Based on these aspects, the experiments were conducted in middle-age/aging C57 black mice in an attempt to model new-onset TLE as seen in adult/aging populations (Ferlazzo et al., 2016). Moreover, the extended hippocampal kindling model and the continuous video/EEG monitoring method were chosen due to previous experience in our lab (Jeffrey et al., 2014; Bin et al., 2017; Stover et al., 2017). In the case of the AEDs, we had to apply the drugs via acute intra-peritoneal injections at anticonvulsive doses as a proof-of-principle test due to the lack of a method for chronic AED delivery in mice in our lab. The three AEDs tested in this study are: phenytoin (Na^+^ channel blocker), lorazepam (benzodiazepine GABA enhancer), and levetiracetam (synaptic vesicle glycoprotein targeting agent). All with different action mechanisms and anti-seizure effects in classic kindling and other models (Löscher et al., 2016; Löscher, 2017).

Our present experiments were aimed to develop a mouse model of extended hippocampal kindling, monitor and assess the SRS stability in individual mice after termination of the kindling stimulation, and evaluate how the three AEDs chosen affect the SRS, the EEG, and the motor behavior.

## Methods

### Animals

Six to 8 months old male C57 black mice were obtained from Charles River Laboratory (C57BL/6N; Saint-Constant, Quebec, Canada) and housed in a local vivarium for several months before experimentation. The vivarium was maintained between 22–23°C and with a 12-h light on/off cycle (light-on starting at 6:00 am). Mice were placed in standard cages with food and water *ad libitum*. We used male mice to avoid variable female sex hormones on kindling process. All experimentations conducted in the present study were reviewed and approved by the Animal Care Committee of the University Health Network in accordance with the Guidelines of the Canadian Council on Animal Care.

### Drugs

Phenytoin and lorazepam were obtained in clinically injectable forms (Sandoz Canada Inc.; Boucherville, Quebec, Canada) while levetiracetam was obtained as a powder from Sigma-Aldrich (Oakville, Ontario, Canada). Phenytoin was stored at room temperature and lorazepam was kept in a fridge at 2–4°C prior to usage. Levetiracetam was dissolved in distilled water as a stock solution and stored in a freezer at −20°C before usage. All three AEDs were appropriately diluted with saline and applied via intra-peritoneal injections (~0.25 ml per injection). Doses were as follows: 25 mg/kg for phenytoin, 1.5 mg/kg for lorazepam and 100 or 400 mg/kg for levetiracetam.

AED injections were made during the light-on period predominantly between 11 AM to 4 PM as SRS were more frequent in late afternoon to evening periods. Injections of different AEDs in each mouse were made ≥ 3 days apart to minimize overlapped drug effects. The AEDs protocol assessments were as follows:

1. Phenytoin. Applied at 25 mg/kg and analyzed its effects in a 10-h period post-injection. This protocol was based on the phenytoin doses used in other mouse models (20–50 mg/kg; Riban et al., 2002; Klein et al., 2015; Twele et al., 2015; Bankstahl et al., 2016) and the half-life of phenytoin plasma elimination in rodents (8–16 h; Löscher, 2007; Markowitz et al., 2010).
2. Lorazepam. Applied at 1.5 mg/kg, similar to the dose range used in rat models of status epilepticus (0.33–4 mg/kg; Walton and Treiman, 1990; Gersner et al., 2016; Kienzler-Norwood et al., 2017). In rats submitted to intravenous injections of this drug (0.9 or 3.2 mg/kg), the half-life of lorazepam plasma elimination is about 2 h. However, lorazepam's brain concentration keeps higher than plasma levels for at least 6 h post-injection (Greenblatt and Sethy, 1990). The latter phenomenon could be related to a more persistent anticonvulsive effect of lorazepam relative to diazepam (Walton and Treiman, 1990; Alldredge et al., 2001). Based on the above information, we used lorazepam instead of diazepam and analyzed its effects in a 10-h period post-injection.
3. Levetiracetam. Applied at 100 mg or 400 mg/kg, similar to the doses used in other rodent models (160–800 mg/kg; Löscher et al., 1993; Löscher and Hönack, 2000; Zhang et al., 2003; Ji-qun et al., 2005; Lee et al., 2013; Shetty, 2013; Twele et al., 2015; Duveau et al., 2016). In mice submitted to intra-peritoneal injections at 200 mg/kg, the half-life of levetiracetam plasma elimination is about 1.5 h and its brain-to-blood ratio is 0.8 at 4 h post-injection (Benedetti et al., 2004; Markowitz et al., 2010). In order to maintain the anti-seizure actions of levetiracetam over a period of time comparable to that of phenytoin or lorazepam, we adapted a three-injection protocol (Löscher and Hönack, 2000). This way, we applied levetiracetam every 4 h at 100 or 400 mg/kg per injection. The effects of levetiracetam were analyzed in a 12-h period following injections.
4. Data similarly analyzed following saline injections or in the next day post AED injection were used to control injection effects and determine recovered SRS.

**Figure 1:**
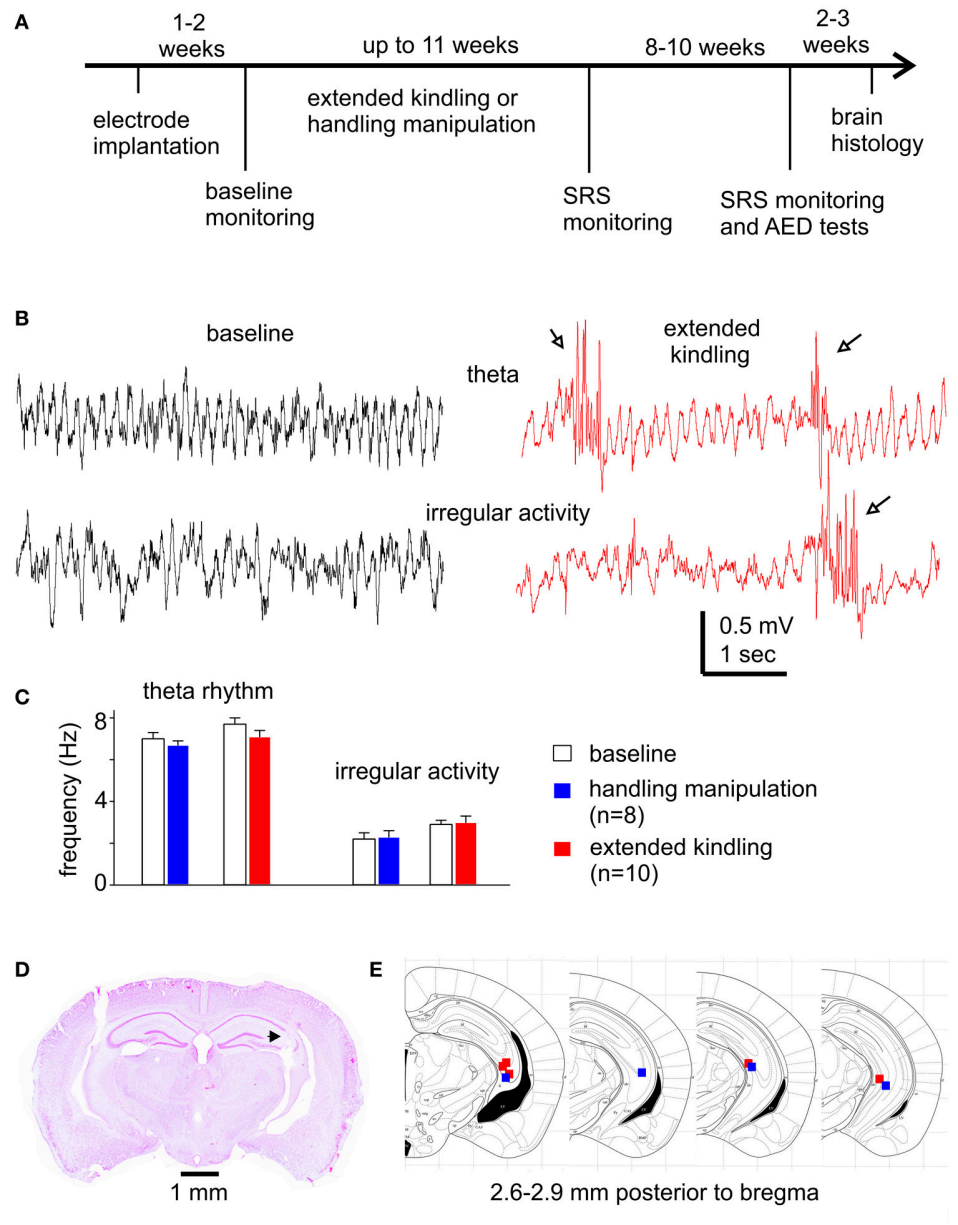
Experimental outline and diverse assessments of the implanted hippocampal electrodes**. (A)** Schematic diagram of the experiments. See text for details. **(B)** Representative hippocampal EEG traces collected from a mouse during baseline monitoring and following extended hippocampal kindling. Arrows show interictal spikes. **(C)** Frequencies of theta rhythms and irregular activities from mice in the control and the extended hippocampal kindled groups. **(D)** Verification of deep electrode site. Image obtained from a mouse following extended hippocampal kindling. Track denoted by a filled arrow. **(E)** Schematic illustrations correspond to coronal brain sections between 2.6 and 2.9 mm posterior to bregma. The solid squares indicate the correct localization of the electrode tips (blue represents 4 control animals and red represents 5 extended hippocampal kindled animals).

The AEDs effects on SRS were examined in extended hippocampal kindled mice (15–17 months old), which might correspond to humans of ≥ 40 years-old (Flurkey et al., 2007). Therefore, we used this experimental paradigm in an attempt to model new-onset TLE seen in adult/aging populations (Ferlazzo et al., 2016). A schematic outline of our experimental procedures is shown in Figure 1A.

### Electrode implantation

Electrodes were constructed in our lab as previously described (Jeffrey et al., 2014; Bin et al., 2017). All electrodes were made of polyamide-insulated stainless steel wires (100 or 230 μm outer diameter; Plastics One, Ranoake, VA). The wires with 100 μm diameter were used in most of experiments to minimize electrode-related tissue injury/perturbations. Twisted bipolar electrodes were used for stimulation and recording (Jeffrey et al., 2014; Stover et al., 2017). For local differential recordings, the two open ends of each implanted bipolar electrode were connected to two inputs of an amplifier headstage (see below and Figure 1 of Bin et al., 2017). For mono-polar recordings, one open end of the bipolar electrode was connected to the amplifier headstage and another open end was grounded as per the instruction of the amplifier manufacturer.

Twenty-six mice were used in the present experiments. These animals were operated for intracranial electrode implantations at ages 11–13 months and then randomly selected for kindling (*n* = 18) or control handling (*n* = 8) experiments. Electrode implantation surgeries were performed similarly as previously described (Jeffrey et al., 2014; Bin et al., 2017; Stover et al., 2017). Briefly, mice were anesthetized with isoflurane and placed on a stereotaxic frame. After skin incision and exposure of the skull, small holes were drilled through the skull, and electrodes were inserted in to the brain by micromanipulators. Electrodes were secured on the skull using a glue-based anchoring screw-free method (Jeffrey et al., 2014). Animals were treated with buprenorphine (0.1 mg/kg, s.c.; every 8 h for two days) to relieve post-surgery pain and allowed to recover for 1–2 weeks prior to further manipulation (Figure 1A). There were no noticeable differences in the animals' body weights and atypical behaviors post-electrode implantation in these two groups of mice. The locations of implanted electrodes were verified by hippocampal EEG activities (Figure 1B) and, in some cases, by later histological assessments (Figures 1D,E).

Bipolar electrodes were implanted in bilateral hippocampal CA3 areas, unilateral hippocampal CA3 and parietal cortical areas or unilateral hippocampal CA3 and piriform areas. The stereotaxic coordinates were 2.5 mm posterior, 3.0 mm lateral and 2.5 mm ventral to bregma for the hippocampal CA3; 0.5 mm anterior, 2.0 mm lateral and 0.5 mm ventral to bregma for the parietal cortical area; 0.5 mm posterior, 3.0 mm lateral and 5.0 mm ventral to bregma for the piriform area (Franklin and Paxinos, 1997). A reference electrode was positioned to a frontal area at 1.5 mm anterior, lateral 1.0 mm and ventral 0.5 mm to bregma. We used such electrode implantations in an attempt to monitor discharge spread from the stimulated CA3 to the contralateral CA3 area or to the ipsilateral parietal cortical or piriform area. However, EEG recordings from the parietal cortical or piriform area were often unstable or with poor signal-to-noise ratio likely due to contaminations of implanted electrodes. Due to these complications, only the EEG signals collected from the stimulated hippocampal area were analyzed in the present experiments.

### Hippocampal kindling

A train of stimuli at 60 Hz for 2 s was used for kindling stimulation (Jeffrey et al., 2014; Bin et al., 2017; Stover et al., 2017). Constant current pulses with monophasic square waveforms, pulse duration of 0.5 ms and current intensities of 10–150 μA were generated by a Grass stimulator and delivered through a photoelectric isolation unit (model S88, Grass Medical Instruments, Warwick RI, USA). An ascending series was used to determine the threshold of evoked afterdischarges in individual mice. The afterdischarges were defined as repetitive spike waveforms with amplitudes of ~2 times of background signals and durations of ≥5 s (Reddy and Rogawski, 2010; Jeffrey et al., 2014; Stover et al., 2017). In the ascending series, the stimulation train with incremental current intensities (10 μA per step) was applied every 30 min. The lowest current by which an afterdischarge event of ≥5 s was elicited was considered the afterdischarge threshold. Stimulations on subsequent days used a stimulation current intensity at 25% above the threshold value (Reddy and Rogawski, 2010). Our attempt was to keep constant stimulation intensity throughout the extended kindling period. However, the initial stimulation intensity often became inconsistent in evoking afterdischarges and motor seizures after ≥ 45 days of kindling experiments, which might be largely due to contaminations of implanted electrodes. Due to this this complication, stronger stimulation intensities (40–80 μA above the initial afterdischarge threshold) were used in subsequent experiments.

A protocol with twice daily stimulations and an inter-stimulation interval of ≥ 5 h, as used in a rat model of extended amygdala kindling (Sayin et al., 2003; Brandt et al., 2004), was adapted for extended hippocampal kindling in the present experiments. Kindling stimulations were conducted in the light-on period between 10 AM and 5 PM. Each stimulation episode lasted 3–5 min, during which the mouse was placed in a bowl-shaped glass container or a large glass beaker for video/EEG monitoring (Stover et al., 2017). All mice in the extended kindling cohort were stimulated for up to 70 consecutive days except 3–7 days for SRS detection (see below). Age-matched control mice received similar electrode implantations and experienced twice daily handlings for 60 consecutive days. During the handling manipulation, each mouse was held for about 1 min and then placed into a large glass beaker for 2–3 min to simulate the stress related to the electrode connection and kindling procedure.

Evoked motor seizures were scored as per the Racine scale (Racine, 1972) modified for mice (Reddy and Rogawski, 2010). Briefly, stage 0 - no response or behavioral arrest; stage 1 - chewing or head nodding; stage 2 - chewing and head nodding; stage 3 - single or bilateral forelimb clonus; stage 4 - bilateral forelimb clonus and rearing; stage 5 - rearing and falling with forelimb clonus.

### EEG recordings and video monitoring

Local differential recordings via twisted bipolar electrodes were used to sample local EEG signals as previously described (Jeffrey et al., 2014; Bin et al., 2017; Stover et al., 2017). Mono-polar EEG recordings were used if local differential recordings were unsuccessful due to electrode contaminations. EEG signals were collected using two-channel or one-channel microelectrode AC amplifiers with extended head-stages (model 1800 or 3000, A-M systems, Sequim, WA, USA). The afterdischarge ipsilateral to the stimulated hippocampus was captured using the model 3000 amplifier via TTL-gated switches between recording and stimulating modes. Signals were collected in a frequency band of 0.1–1,000 or 0.1–3,000 Hz and amplified 1,000 × before digitization (5,000 or 1,0000 Hz, Digidata 1440A or 1550, Molecular Devices; Sunnyvale, CA, USA). Data acquisition, storage and analyses were done using pClamp software (Version 10; Molecular Devices).

Continuous video/EEG monitoring (roughly 24 h daily for several consecutive days) was used in the morality of experiments to detect SRS (Bin et al., 2017). Mice were placed in a modified cage with food and water available *ad libitum*. A webcam was placed near the cage to capture the animals' motor behaviors. EEG and video data were saved every 2 h using a Mini Mouse Macro program. Dim lighting was used for webcam monitoring in the light-off period. Data collection was stopped for ~30 min daily for animal care. Intermittent EEG recordings during the light-on period (4–8 h per session) were used in some of baseline monitoring experiments. For such recordings, each mouse was placed into the glass container and allowed to freely access hydrogel and food pellets provided on floor (Stover et al., 2017).

Baseline monitoring was conducted 1–2 weeks post-electrode implantation (Figure 1A). For each mouse following extended kindling, continuous video/EEG monitoring of 24–72 h per session was conducted after the 80, 100, 120, and/or 140th stimulation to assess SRS commencement. If ≥ 2 SRS events were observed in 24 h, no further stimulation was applied and additional video/EEG monitoring for up to 72 h was performed to assess initial SRS incidences. Video/EEG monitoring of 24–72 h was repeated 8–11 weeks later to assess SRS stability. The effects of AEDs were examined afterwards. For each mouse in the control group, continuous video/EEG recordings of 48 h were performed after 60 days of twice daily handling manipulations.

### Brain histological assessments

Brain histological sections were prepared using a protocol modified from our previous studies (Jeffrey et al., 2014; Stover et al., 2017). Mice were anesthetized via sodium pentobarbital (100 mg/kg, intra-peritoneal injection) and perfused trans-cardiacally with saline and then with 10% neutral buffered formalin solution (Sigma-Aldrich; Oakville, Ontario, Canada). Removed brains were further fixed in a hypertonic (with 20% sucrose) formalin solution. Coronal sections of 50 μm thick were obtained using a Leica CM3050 research cryostat and placed onto glass slides (Superfrost plus microscope slides, Fisher Scientific, Canada). Brain sections were dried in room air for ≥1 week, processed sequentially with chloroform (24 h), 95% ethanol (24 h), 90% ethanol (12 h), and 70% ethanol (0.5 h), and then stained with cresyl violet (0.1%, Sigma Aldrich, Oakville, Ontario, Canada). Images of brain sections were obtained using a slide scanner (Aperio digital pathology slide scanner AT2, Leica) at 20 × magnification and analyzed using ImageScope (Leica) or Image J (National Institute of Health, USA) software.

### Data analysis

Spectral analysis was used to determine the main frequencies of hippocampal rhythms. Spectral plots (rectangular function, 50% window overlap and spectral resolution 0.3 Hz; PClamp software) were generated from 60- to 15-s data segments that encompassed theta rhythmic or irregular signals. Three spectral plots were averaged to assess baseline and post-kindling or post-handling EEG signals in each mouse.

Interictal EEG spikes were recognized by intermittent events with large peak amplitudes (≥6 times of standard deviation of background signals), simple or complex spike waveforms, and durations of 30–250 ms. Spike incidences were measured from the stimulated hippocampal area and in 10-min data segments collected during immobility/sleep as interictal spikes manifested in these “inactive” behavioral states. These data were collected ≥30 min after a preceding ictal event to avoid the influences of ictal discharges on subsequent interictal spikes. An event detection function (threshold search method) of pClamp software was used to detect spikes automatically, and detected events were then visually inspected and false events were rejected.

SRS were determined by EEG ictal discharges of ≥30 s in durations and concurrent motor seizures of stages 2–5 according to the Racine scale modified for mice (Racine, 1972; Reddy and Rogawski, 2010). In some experiments, SRS were determined by EEG ictal discharges alone due to errors in video acquisition/storage. In our present experiment, each mouse was monitored by a webcam from a side of its housing cage. This setting was inadequate to capture stage 2–4 motor seizures if the mouse's head and forelimbs were not faced toward the webcam. Due to this complication, we did not attempt to detect SRS by motor behaviors alone.

### Statistical analysis

A Student's *t*-test or Mann-Whitney Rank Sum Test was used for two-group comparisons (Sigmaplot; Systat Software Inc., San Jose, California, USA). A one-way ANOVA followed by a multiple comparison test (Dunn's or Holm-Sidak method) was used for multiple group comparisons. Mean and standard error of the mean (SEM) were presented throughout the text and figures. Statistical significance was set at *p* < 0.05.

## Results

Twenty-six mice were used in the present experiments (*n* = 18 for kindling and *n* = 8 for control handling). Of the 18 mice in the kindling group, 14 were “kindled” following 25–35 stimulations as they exhibited 3 consecutively evoked stage-5 motor seizures (Reddy and Rogawski, 2010; Jeffrey et al., 2014; Stover et al., 2017); other 4 mice were excluded after several days of kindling experiments due to inconsistence/failure in evoking afterdischarges. The 14 “kindled” mice were used for extended kindling. SRS induction was successful in 12 mice following ≥ 100 stimulations (Table 1); the remaining 2 mice were euthanized after the 45 or 70th stimulation due to a loss of implanted electrodes or severe skin lesion. In the mice that exhibited SRS following extended kindling, the mean durations of evoked hippocampal afterdischarges were 17.5 ± 2.1, 22.4 ± 1.4, 25.4 ± 3.1, 27.9 ± 1.0, and 31.9 ± 3.1 s in response to 1–20, 21–40, 41–60, 61–80, and 81–100 stimuli, respectively. The mean stages of corresponding motor seizures were 3.7 ± 0.6, 4.5 ± 0.3, 4.1 ± 0.3, 4.1 ± 0.2, and 4.0 ± 0.3 respectively. The afterdischarges evoked by 61–80 and 81–100 stimuli were significantly longer than that evoked by the first 20 stimuli (*p* ≤ 0.05); whereas there were no significant differences among corresponding motor seizure stages. Cumulative afterdischarge durations or motor seizure stages to SRS were variable in these mice, ranging from 2,311 to 3,009 s or from 343 to 449. In general, all these observations are in accordance with the previous study done in a rat model of extended amygdala kindling (Brandt et al., 2004). However, data from a larger cohort of mice are needed to delineate the relationship between evoked seizures and induced SRS in our model.

**Table 1:** Mice examined in the present experiments.

**Mouse number/total stimuli applied to achieve extended kindling**	**SRS events/monitoring times**		**AEDs tested in each mouse**
	**Initial^#^ (h)**	**Later^$^ (h)**	
#1, 120	7/48	4/24	PHE^25mg/kg^
#2, 140	13/72	16/144	PHE^25mg/kg^
#3, 140	22/48	12/48	PHE^25mg/kg^, LEV^100mg/kg^
#4, 100	9/48	17/72	PHE^25mg/kg^, LEV^100mg/kg^
#5, 100	10/48	5/40	PHE^25mg/kg^, LEV^100mg/kg^
#6, 100	11/66	6/24	PHE^25mg/kg^, LEV^100mg/kg^
#7, 140	23/72	26/93	LOZ^1.5mg/kg^, LEV^100, 400mg/kg^
#8, 120	20/72	20/72	LOZ^1.5mg/kg^, LEV^100, 400mg/kg^
#9, 120	17/72	10/24	LOZ^1.5mg/kg^, LEV^400mg^
#10, 120	9/48	17/72	LOZ^1.5mg/kg^, LEV^400mg^
#11, 120	7/48	4/24	LOZ^1.5mg/kg^, LEV^400mg^
#12, 120	13/72	16/144	LOZ^1.5mg/kg^, LEV^400mg^
**Mouse Number/totalandlings Applied**			
#1-#8, 120	0/48 h		

PHE, phenytoin; LOZ, lorazepam; LEV, levetiracetam;

# and $ *individual mice were monitored initially within 3 days after termination of kindling stimulation; then, monitored 8–11 weeks later. Control mice were monitored after 120 handling manipulations*.

### Hippocampal EEG rhythms and brain histological observations

The mouse hippocampus is known to exhibit the theta rhythm (5–12 Hz) during movement/exploration and irregular activity (0.5–4 Hz) during immobility/sleep (Buzsáki et al., 2003). We examined these EEG signals to explore potential alterations by extended kindling and to electrographically verify the locations of implanted hippocampal electrodes. Hippocampal theta and irregular activities were consistently observed from mice in the kindling and control handling groups (Figure 1B). In the kindling group (*n* = 10), the frequencies of theta rhythm and irregular activity were 7.7 ± 0.3 and 2.9 ± 0.2 Hz during baseline monitoring and 7.1 ± 0.3 and 3.0 ± 0.3 Hz following 100–120 hippocampal stimulations. In the control group (*n* = 8), the frequencies of theta rhythm and irregular activity were 7.0 ± 0.3 and 2.2 ± 0.3 Hz during baseline monitoring and 6.7 ± 0.2 and 2.3 ± 0.3 Hz following 120 handling manipulations (Figure 1C). There were no significant differences between group measures or between baseline and post-stimulation or post-handling measures (*p* ≥ 0.207). However, frequent interictal spikes were consistently observed in mice following extended kindling (Figure 1B). Further analysis are needed are needed to reveal whether these hippocampal rhythms are altered by extended kindling in other biophysical domains.

Brain histological sections were prepared from 10 mice that underwent extended kindling or handling manipulations (*n* = 5 in each group). These sections were stained with cresyl violet for general assessments of gross brain lesion and the tracks of implanted hippocampal electrodes. There were no evident gross brain lesions, such as structural deformation, cavity, or dark-stained scar tissues, in these kindled or control mice examined. However, detailed histological assessments are needed to reveal a potential loss of hippocampal GABAergic interneurons in our model (Sayin et al., 2003; Brandt et al., 2004). Of the 10 mice examined, the tracks of implanted hippocampal electrodes were evident in 5 kindled and 4 control mice. These tracks were recognizable in sections (2.6–2.9 mm posterior to bregma) appropriate to the stereotaxic coordinates of designated hippocampal CA3 area (Figures 1D,E). These histological observations together with the above EEG measures suggest that targeting the mouse hippocampus for extended kindling was generally reliable in our present experiments.

### Main features of SRS

Continuous (≥24 h) video/EEG monitoring was used to detect SRS in our present experiments. Such monitoring was conducted after the 80, 100, 120, and/or 140th stimulation to assess SRS commencement. SRS were consistently observed following 100–140 hippocampal stimulations (*n* = 12; Table 1) but were undetectable or with very low incidences following the 80th stimulation. Neither SRS nor interictal spikes were observed in the age-matched controls (*n* = 8) when examined after 120 handling manipulations (Table 1). Together these observations suggest that SRS genesis may require sufficient accumulation of evoked seizures and that the stress imposed by chronic handlings per se is not a causal factor of SRS.

**Figure 2:**
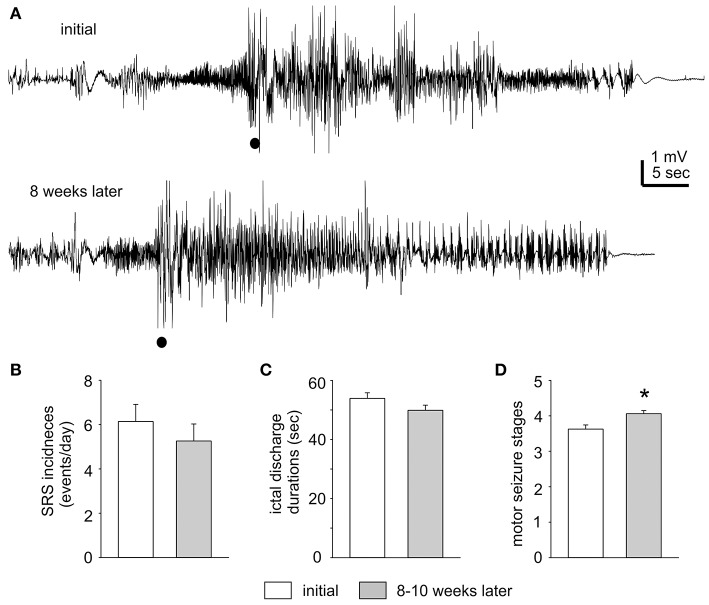
Stable SRS observed after termination of kindling stimulation. **(A)** The first EEG trace corresponds to a mouse on Day 1 after termination of extended hippocampal kindling. The second trace shows the EEG of the same animal but 8 weeks later. Filled circles denote movement artifacts. **(B**–**D)** SRS incidences, corresponding hippocampal discharge duration, and motor seizure stages measured within 3 days after termination of extended hippocampal kindling stimulation and 8–11 weeks later (*n* = 10). The values represent the mean ± S.E.M. *p = 0.002.

SRS were recognized by hippocampal ictal discharges and concurrent motor seizures. These discharges displayed a low-voltage fast onset (Lévesque et al., 2012), repetitive spike waveforms lasting tens of seconds and a postictal EEG suppression of a few seconds (Figure 2A). Such discharge waveforms were consistently observed in all 12 mice over a few months after termination of kindling stimulation. This phenomenon suggests that a similar epileptic network activity may underlie these discharges. EEG ictal discharges were also observed from the parietal cortical or piriform area in some experiments (data not shown). The motor seizures corresponding to the EEG discharges were featured predominantly with forelimb clonus, rearing and/or falling, which corresponded stage 3–5 seizures according to the Racine scale modified for mice (Supplemental Videos 1-2). Together these EEG and motor behavioral observations are indicative of SRS as generalized seizure events.

The incidence and severity of SRS were relatively stable in individual mice examined. Measured in 1–3 days after termination of kindling stimulation and 8–11 weeks later, SRS incidences were 6.1 ± 0.8 and 5.2 ± 0.8 events/day; corresponding hippocampal discharge durations were 53.9 ± 1.9 and 49.9 ± 1.7 s and motor seizure stages were 3.6 ± 0.1 and 4.1 ± 0.1 respectively (*n* = 10; Figures 2B–D). There were no significant time-dependent changes in the SRS incidences and discharge durations (*p* = 0.43 and 0.133), but a slight increase in motor seizure stages (*p* = 0.002). Examples of stable SRS are presented in Figure 2A and Supplemental Videos 1–2, where similar hippocampal discharges and corresponding stage-5 motor seizures were recorded from a mouse in 24 h after termination of kindling stimulation and about 8 weeks afterwards.

### AED effects on SRS

**Figure 3:**
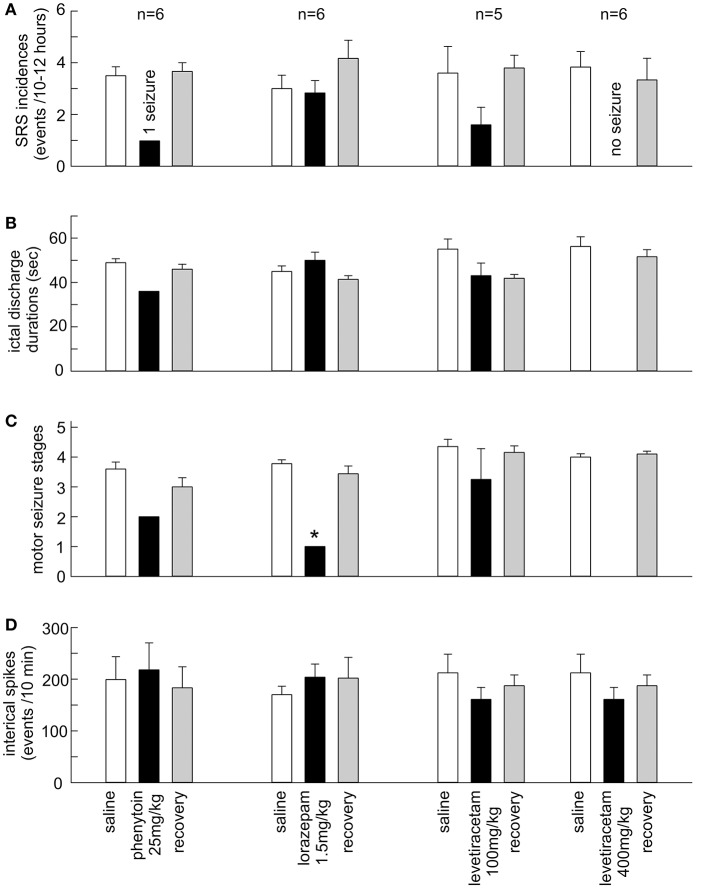
Effects of the AEDs on SRS and hippocampal interictal spikes. **(A**–**D)** Phenytoin (25 mg/kg), lorazepam (1.5 mg/kg) and levetiracetam (100 and 400 mg/kg) were applied via intra-peritoneal injections. SRS were measured 10–12 h post saline or AED injection. Interictal spikes were measured ~1 h post saline or AED injection. Recovered measures were done 24 h post AED injection. The values represent the mean ± S.E.M. *p = 0.002.

Phenytoin effectively suppressed SRS in 6 mice tested. SRS incidences were 3.5 ± 0.3 events/10 h following saline injections. No SRS were detected in 5/6 mice and only 1 SRS event was observed in another mouse following phenytoin injections (*p* = 0.002; Figures 3A–C). While hippocampal ictal discharges were nearly abolished by phenytoin, hippocampal interictal spikes persisted without evident changes in waveforms and incidences. Measured at 1 h post saline or phenytoin injection, the incidences of hippocampal interictal spikes were 199.2 ± 44.2 and 218.0 ± 55.2 spikes/10 min, respectively (*n* = 6 mice; *p* = 0.789; Figure 3D). Hippocampal EEG signals collected from a mouse are illustrated in Supplementary Figure 1, showing interictal spikes and a subsequent discharge event following a saline injection and interictal spikes alone after a phenytoin injection.

Levetiracetam at 400 mg/kg abolished SRS in 6/6 mice tested. SRS incidences were 3.8 ± 0.6 events/12 h following saline injections. Neither hippocampal discharges nor motor seizures were detected following levetiracetam injections (Figures 3A–C), whereas the incidences of hippocampal interictal spikes were not significantly different between post-saline and post-levetiracetam measures (212.3 ± 35.9 and 160.8 ± 23.2 events/10 min; *p* = 0.123; Figure 3D). Levetiracetam at 100 mg/kg was variable in suppressing SRS (*n* = 5 mice). SRS incidences were decreased in 4/5 mice tested but the overall SRS incidences were not significantly different between post-saline and post-levetiracetam measures (3.6 ± 1.0 vs. 1.6 ± 0.7 events/12 h; *p* = 0.151; Figure 3A). There were no significant differences between post-saline and post-levetiracetam measures about corresponding hippocampal discharge durations (55.0 ± 4.6 vs. 43.1 ± 5.7 s; *p* = 0.134), motor seizure stages (4.4 ± 0.2 vs. 3.3 ± 1.0; *p* = 0.489) and hippocampal interictal spikes (227.2 ± 51.6 vs. 171.8 ± 42.3 events/10 min; *p* = 0.430; Figures 3B–D).

**Figure 4:**
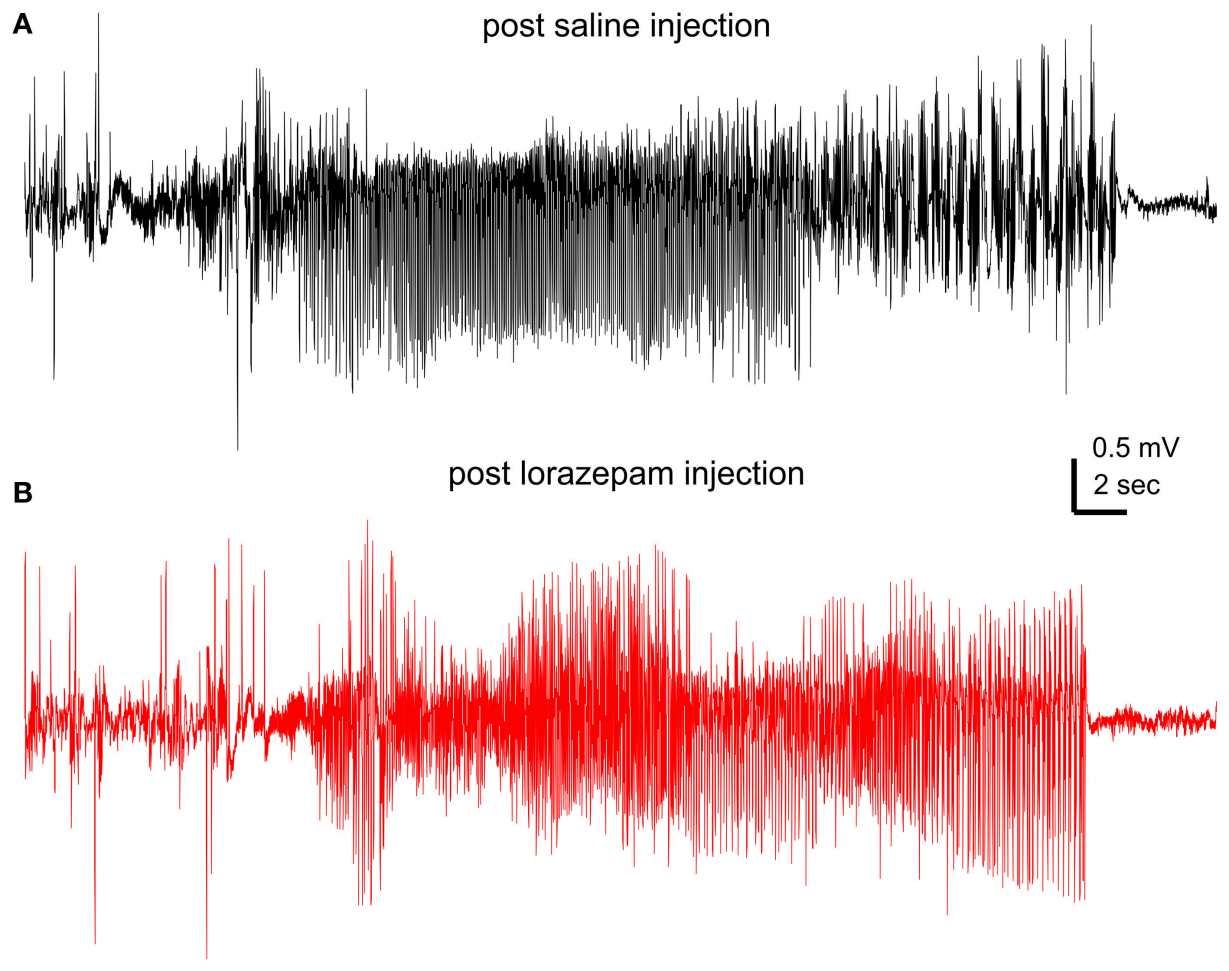
Effects of lorazepam on hippocampal EEG discharges. EEG traces collected from a mouse following a saline **(A)** or a lorazepam (1.5 mg/kg) **(B)** injection. Corresponding motor seizures presented in Supplementary Videos 3, 4.

Lorazepam at 1.5 mg/kg effectively suppressed motor seizures but spared corresponding hippocampal discharges in 6 mice examined. SRS incidences were not significantly different between post-saline and post-lorazepam measures (3.0 ± 0.5 and 2.8 ± 0.5 events/10 h, *p* = 0.234). Motor seizure stages were significantly decreased from 3.8 ± 0.1 following saline injections to 1.0 in all 6 mice following lorazepam injections (*p* = 0.002); whereas the durations of corresponding hippocampal discharges were not significantly different between post-saline and post-lorazepam measures (45.0 ± 2.5 and 50.0 ± 3.7 s; *p* = 0.191; Figures 3A–C). The incidences of hippocampal interictal spikes were also not different between post-saline and post-lorazepam measures (170.0 ± 16.2 and 203.8 ± 25.3 events/10 min; *p* = 0.656; Figure 3D). SRS sampled from a mouse are presented n in Figure 4 and Supplementary Videos 3-4, showing two similar hippocampal discharges in correspondence to a stage-4 following a saline injection and a stage-1 motor seizure following a lorazepam injection.

## Discussion

We aimed to develop a mouse model of extended hippocampal kindling with a particular focus on AED effects in the present study. Three main observations emerged from our present study. First, extended hippocampal kindling and chronic SRS monitoring were reliable. Second, SRS manifested as generalized seizure events with relatively stable incidence and severity. Third, SRS responded differently to phenytoin, lorazepam, and levetiracetam according to EEG and motor behavioral measurements. These observations suggest that the extended hippocampal kindling could be considered as a mouse model of SRS.

### Extended hippocampal kindling and SRS induction

Reliable stimulation and recording of a desired brain structure are essential for extended kindling in mice. To address this issue, we used our group's previously developed methods in order to achieve stable implantations of intracranial electrodes and chronic EEG monitoring in mice (see Jeffrey et al., 2014 and Bin et al., 2017 for details). To verify the locations of the implanted hippocampal electrodes, we examined the theta rhythm and the irregular activity as they are intrinsic, behavioral state-dependent hippocampal activities (Buzsáki et al., 2003). Both hippocampal activities were consistently observed in all mice examined during baseline monitoring and following extended kindling or handling manipulation. We also performed brain histology to reveal the tracks of implanted hippocampal electrodes. This evaluation showed appropriate electrode placements and no gross brain lesions in the majority of the examined animals. These results support the idea that extended hippocampal kindling is in general a feasible and reliable model. However, extended kindling of other brain structures in mice remains to be tested.

### SRS following extended hippocampal kindling

In our present experiments, SRS were recognized by hippocampal EEG ictal discharges and concurrent motor seizures. EEG ictal discharges were also observed from the parietal cortical or piriform area in some experiments. As the hippocampus is not directly involved in motor functions, we speculate that the hippocampal discharges may be a major component of focal seizures and that generalized motor seizures may be a result of seizure spread from the hippocampus to other brain areas. Regarding the EEG and the motor behavioral features of the SRS that had the animals, our observations are in line with previous studies of extended kindling in other animal species (Wada et al., 1974; Wada and Osawa, 1976; Pinel and Rovner, 1978; Wauquier et al., 1979; Gotman, 1984; Hiyoshi et al., 1993; Milgram et al., 1995; Michalakis et al., 1998; Sayin et al., 2003; Brandt et al., 2004). However, some important features such as the SRS development, the regional EEG discharges, the neuronal injury, and the hippocampal cellular and local circuitry activities (Sayin et al., 2003; Brandt et al., 2004) need to be characterized in our model.

To assess SRS stability, individual mice were monitored over a few months after termination of the kindling stimulation. These experiments revealed that SRS were relatively stable in incidence, hippocampal discharge durations and motor seizure stages. Overall, the mean SRS incidences in individual mice were in a range of 4–6 events/day, which appear to be higher than those monitored via continuous video/EEG in a rat model of extended amygdala kindling (1–2 SRS events/day; Brandt et al., 2004). While such a difference may be due to multiple experimental variables including animal species, ages and kindling sites, SRS with a relatively stable and high incidence are a significant outcome of our present experiments.

### SRS following extended hippocampal kindling are suitable for AED assessments

#### AED effects and potential anti-seizure mechanisms

Phenytoin and levetiracetam at 400 mg/kg abolished SRS in the majority of mice examined as neither hippocampal discharges nor motor seizures were detected in these mice following AED injections. Levetiracetam at 100 mg/kg was inconsistent in suppressing SRS, which might be due in part to a small tested sample size and to dose-dependent effects. In the case of lorazepam at 1.5 mg/kg, it differently affected the SRS when compared to phenytoin and levetiracetam (400 mg/kg); it decreased motor seizure stages while no significant reductions were observed in SRS incidence and hippocampal discharge durations.

Previous studies have examined anti-seizure effects of phenytoin, diazepam and levetiracetam in classic kindling models. The suppression of evoked focal seizures, by increased afterdischarge threshold and/or decreasing afterdischarge durations, is thought to be the primary target for phenytoin's anticonvulsant action (Ebert et al., 1997). Levetiracetam also increases afterdischarge thresholds or decreases afterdischarge durations (Löscher et al., 1998, 2016; Löscher and Hönack, 2000). Benzodiazepine GABA enhancers such as diazepam and clonazepam at low doses can decrease generalized motor seizures with or without weak effects on evoked focal afterdischarges (Löscher et al., 1986; Voits and Frey, 1994). Our present observations may be in line with these previous findings if spontaneous hippocampal discharges represent a major component of focal seizures and motor seizures result from seizure spread from hippocampus to other brain areas in our model.

#### Comparisons with relevant previous studies

A previous study has tested phenytoin and diazepam in a rat model of extended amygdala kindling (Pinel, 1983). In this study, phenytoin at 100 mg/kg and diazepam at 1 mg/kg were administered repeatedly by daily intra-peritoneal injections, and AED effects on spontaneous motor seizures (mainly forelimb clonus) were examined in a 3-h period post-injection. Phenytoin effectively suppressed the spontaneous motor seizures whereas diazepam had litter effects. This previous study and our present experiments are in agreement regarding the effects of phenytoin, suggesting that this AED may be effective in controlling SRS in extended kindling models. The ineffectiveness of diazepam (1 mg/kg) seen in the rat model differs from the motor seizure suppression induced by lorazepam (1.5 mg/kg) in our model.

Several studies have examined phenytoin, diazepam and/or levetiracetam in a mouse model of intra-hippocampal kainate application (Riban et al., 2002; Klein et al., 2015; Twele et al., 2015; Duveau et al., 2016). In these studies, AEDs were applied by acute intra-peritoneal injections and their effects on hippocampal EEG discharges were examined in periods of 3-4 h post-injection. Diazepam at 2–5 mg/kg and levetiracetam at 600 or 800 mg/kg (but not at 200 or 400 mg/kg) were effective in suppressing hippocampal discharges; whereas phenytoin at 20–50 mg/kg was ineffective. Discrepancies appear to exist between these findings and our present observations on the effects of phenytoin and diazepam/lorazepam as well as on the anti-seizure doses of levetiracetam. However, mouse strains, ages, seizure types and AED administration and assessment protocols are also different between these studies and our experiments. Of these experimental dissimilarities, distinct seizure types and underlying epileptogenic processes are likely major influencing factors of model-specific AED effects (Löscher, 2017).

### Significance and other limitations

Many patients with TLE do not present major structural abnormalities in standard brain imaging examinations (Ferlazzo et al., 2016). In addition, status epilepticus is not usually recognized in these patients in the early phase of epileptogenic process. Therefore, it is important for experimental research to model these clinical scenarios. However, the commonly used status epilepticus models induce evident brain lesions (Dudek and Staley, 2017; Gorter and van Vliet, 2017; Henshall, 2017; Kelly and Coulter, 2017) which may be suboptimal for research along this direction. In this regard, extended kindling may represent an optimal candidate model since the SRS developed are not initiated and/or associated with status epilepticus and gross brain lesions (Pinel and Rovner, 1978; Milgram et al., 1995; Michalakis et al., 1998; Sayin et al., 2003; Brandt et al., 2004). To date, SRS genesis following extended kindling is not fully understood yet, and extended kindling is not widely used mainly due to its laborious nature and the required reliability of implanted intracranial electrodes in chronic experiments. Furthermore, no study involving a mouse model of extended kindling has been published yet. In this sense, our present work is pioneer in the development of a mouse model of extended hippocampal kindling and its validation for future chronic SRS examinations and AED assessments. This model, with further improvement and possible employment of genetically/molecularly manipulated mouse strains, may facilitate future research involving epileptogenesis and drug-resistant epilepsy in the absence of major brain lesions/pathology as seen clinically.

Our present experiments have some limitations. In particular, we did not test each AED at multiple doses nor examine all three AEDs in individual mice. The lack of these experiments disallows AED efficacy assessments in the present study. In addition, EEG recordings from the cortical or piriform area were inconsistent and/or poor in signal quality likely due to electrode contaminations and/or position errors. These drawbacks prevent analyses of regional discharges and their changes by AEDs. Furthermore, the methods/facility for chronic AED delivery (Grabenstatter et al., 2007; Ali et al., 2012) and for measuring AED levels in serum and brain (Löscher, 2007; Markowitz et al., 2010) need to be established for our model, as these approaches are critical for testing AEDs in a clinically relevant manner and for exploring drug-resistant epilepsy. Despite these limitations and weaknesses, our experiences may help future AED assessments in mouse models of extended kindling.

## Author contributions

HS, UT, JC, NS, CC, SL, and CW: contributed to experimentation and data analysis; UT, JF, JE, and LZ: contributed to data analysis, data discussion, and manuscript writing. All authors contributed to experimental design.

### Conflict of interest statement

The authors declare that the research was conducted in the absence of any commercial or financial relationships that could be construed as a potential conflict of interest.

## Supplementary material

Supplementary Figure 1 Effects of phenytoin on hippocampal EEG signals. Continuous EEG signals presented separately for illustrative purpose. **(A,B)**, interictal spikes (top) and subsequent ictal discharge (bottom) observed following a saline injection. **(C,D)**, only interictal spikes observed following a phenytoin injection.

Supplementary Video 1-2 Representative motor seizures. Video data collected from a mouse in the first day (initial) after termination of kindling stimulation and about 8 weeks later. Motor seizures corresponded to the EEG ictal discharges illustrated in Figure 2A top and bottom. Videos trimmed with motor seizures appearing around the 6 s time stamp.

Supplementary Video 3-4 Motor seizure suppression by lorazepam. Motor seizures captured from a mouse following a saline injection (video 3) and a lorazepam injection (video 4). Videos arranged with the motor seizure activities appearing around the 7 s time stamp. Corresponding hippocampal discharges presented in Figures 4A,B.
