# Supplementary figures and images for: Effects of Antiepileptic Drugs on Spontaneous Recurrent Seizures in a Novel Model of Extended Hippocampal Kindling in Mice
Source: Front Pharmacol. 2018 May 18;9:451. doi: 10.3389/fphar.2018.00451 (PMC5968120; doi:10.3389/fphar.2018.00451)

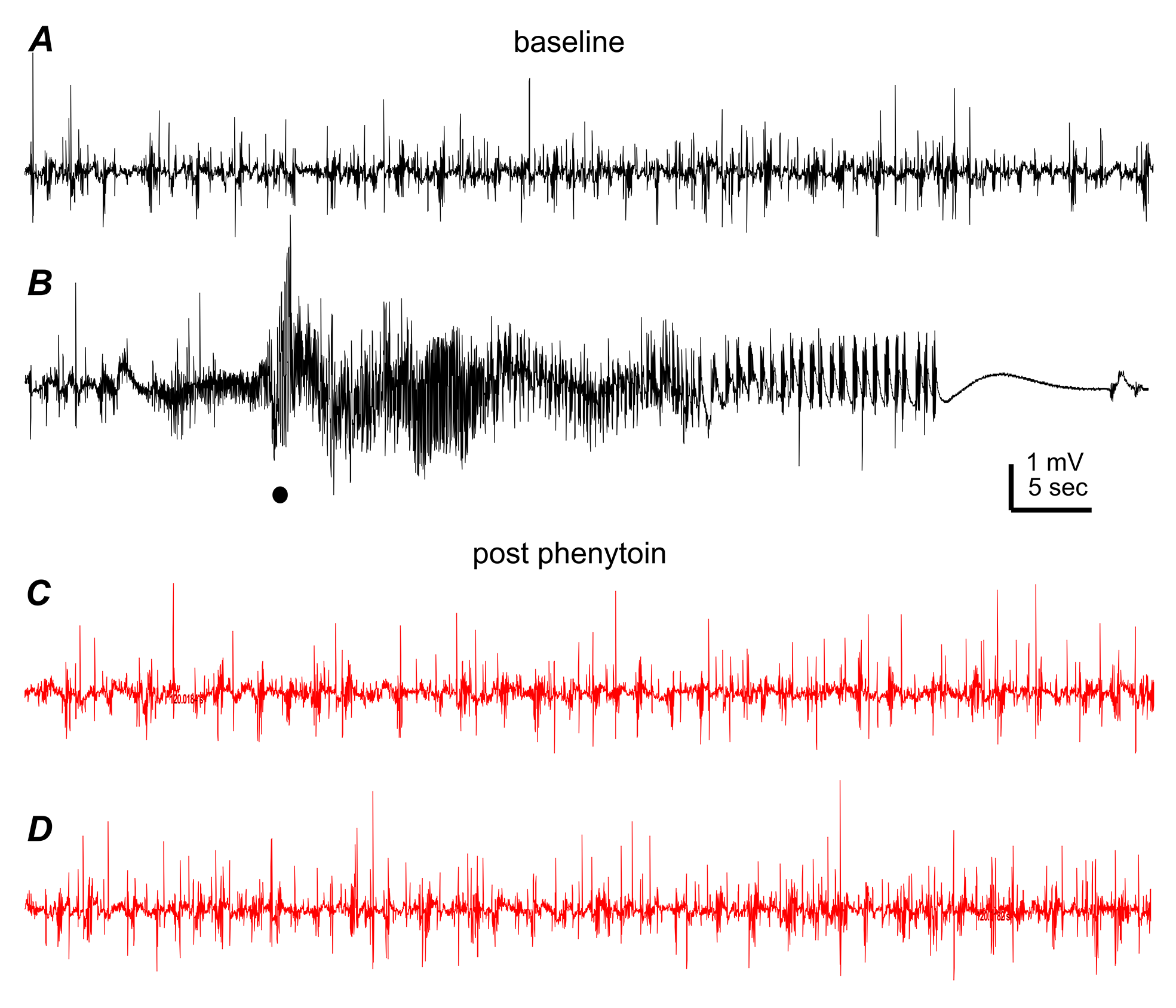

Supplement: Supplementary Figure 1 — Effects of phenytoin on hippocampal EEG signals. Continuous EEG signals presented separately for illustrative purpose. (A,B), interictal spikes (top) and subsequent ictal discharge (bottom) observed following a saline injection. (C,D), only interictal spikes observed following a phenytoin injection. [file Image_1.TIF]
